# MicroRNA profiling identifies MiR-195 suppresses osteosarcoma cell metastasis by targeting CCND1

**DOI:** 10.18632/oncotarget.3560

**Published:** 2015-03-12

**Authors:** Kang Han, Xiang Chen, Na Bian, Baoan Ma, Tongtao Yang, Chengkui Cai, QingYu Fan, Yong Zhou, TingBao Zhao

**Affiliations:** ^1^ Department of Spinal Cord Injury, General Hospital of Jinan Military Area Command of Chinese PLA, Jinan, Shandong, People's Republic of China; ^2^ Department of Baylor College of Medicine, Houston, Texas, USA; ^3^ Department of Orthopedic Surgery, Orthopedics Oncology Institute of Chinese PLA, Tangdu Hospital, Fourth Military Medical University, Xi'an, Shaanxi, People's Republic of China

**Keywords:** microRNA, miR-195, osteosarcoma, CCND1, metastatic

## Abstract

Metastasis is a leading cause of mortality for osteosarcoma patients. The molecular pathological mechanism remains to be elucidated. In the previously study, we established two osteosarcoma cell lines with different metastatic potentials. Differential expressed genes and proteins regarding metastatic ability have been identified. MicroRNAs are important regulators in tumorigenesis and tumor progression. In this study, microRNA microarray was used to assess the differential expressed miRNAs level between these two cell lines. One of the top ranked miRNAs-miR-195 was identified highly expressing in lowly metastatic cells. It was showed that over-expression of miR-195 substantially inhibits migration and invasion of osteosarcoma cells *in vitro* and pulmonary metastasis formation *in vivo*. Meanwhile, CCND1 was identified as the target gene of miR-195 and further studied. More importantly, Using real-time PCR, we evaluated the expression of miR-195 and CCND1 in osteosarcoma samples from 107 frozen biopsy tissues and 99 formalin- or paraformalin-fixed, paraffin-embedded (FFPE) tissues. Results indicated lowly expressed miR-195 or highly CCND1 correlated with positive overall survival and their expression inverse relate to each other. In summary, our study suggests miR-195 function as a tumor metastasis suppressor gene by down-regulating CCND1 and can be used as a potential target in the treatment of osteosarcoma.

## INTRODUCTION

Osteosarcoma (OS) is the most common primary malignant bone tumor in children and adolescents. The estimated incidence rate worldwide is 4/million/year, with a peak incidence at the age of 15–19 years [1]. However, there are no noticeable improvements in term of survival of patients for three decades especially for the subgroup which had initial metastasis at diagnosis [2]. It is very emergent to find the new targets for OS metastasis treatment. MicroRNAs (miRNAs) are 20–22 nucleotide RNA molecules that have been shown to regulate the expression of different genes in a variety of eukaryotic systems [3, 4]. MiRNAs exert at least a part of their biological effects as guides for post-transcriptional gene silencing, producing sequence-specific mRNA cleavage or translational repression that can have dramatic effects on cellular phenotype [5, 6]. Given the fundamental biological processes that are regulated by miRNAs, it has been known miRNAs play an important role in aging, differentiation and cancer [7].

MiR-195 was originally predicted based on homology to a verified miRNA from the mouse [8] and was later shown to exist in humans [9]. Recently, miR-195 has been reported to be deregulated in certain types of cancer, including up-regulation in chronic and acutely mphocytic leukemia [10] and metastatic melanoma [11] but down-regulation in adrenocortical, hepatocellular carcinoma, and squamous cell carcinoma [12-14]. However, the exact role of miR-195 plays in OS remains elusive. CCND1 encodes Cyclin D1 protein which is essential for G1 to S transition. Recently it has been reported that cyclin D1 involve in tumor progression and metastasis [15]. Previous studies have shown that miR-195 prevents cell proliferation and promotes apoptosis in other cancers by binding to the 3′–UTR of mRNAs of CCND1 [16-18]. However, the relationship between the expression of miR-195 and its target gene CCND1, especially the new pro-metastatic role of CCND1, has not been reported in OS.

In this study, we used miRNA microarray to screen the differential expressed miRNAs and found miR-195 highly expressed in lowly metastatic OS cell lines. Further study showed that over-expression of miR-195 inhibited metastasis of OS cells both *in vitro* and *in vivo*. We then demonstrated osteosarcoma samples frequently featured lowly expressed miR-195 comparing to the normal tissue counterparts. Expression of MiR-195 is an independent factor to predict the over-all survival of OS patients. In addition, CCDN1 was identified as the target gene of miR-195 in OS and its encoded protein (CyclinD1) correlate inversely with miR-195. All these results indicate miR-195 and its downstream target gene CCND1 can be used to predict the prognosis or even treat the metastatic OS in the future.

## RESULTS

### MiRNA expression profiling indicates miR-195 highly express in the lowly metastatic OS cell lines

**Figure 1:**
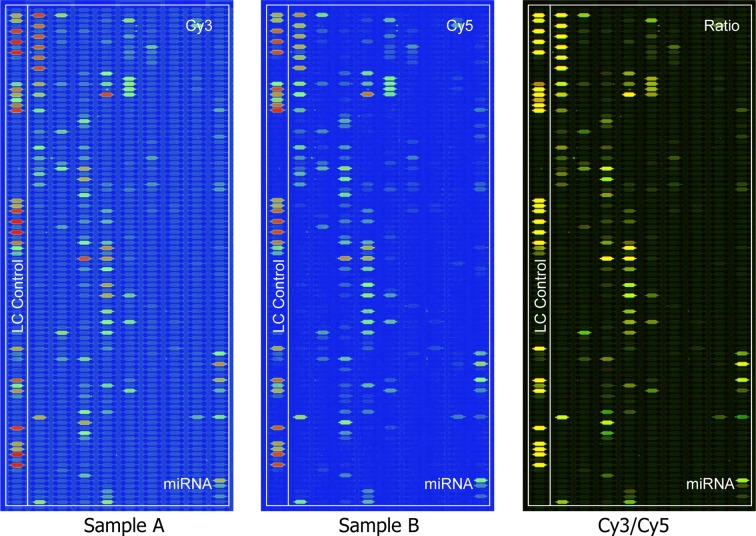
Different microRNA expression between F4 (Labeling Dye: Cy3) and F5M2 (Labeling Dye: Cy5) by the MicroRNA microarray The images are displayed in pseudo colors so as to expand visual dynamic range. In the Cy3 and Cy5 intensity images, as signal intensity increases from 1 to 65,535 the corresponding color changes from blue to green, to yellow, and to red. In the Cy3/Cy5 ratio image, when Cy3 level is higher than Cy5 level the color is green; when Cy3 level is equal to Cy5 level the color is yellow; and when Cy5 level is higher than Cy3 level the color is red.

**Figure 2:**
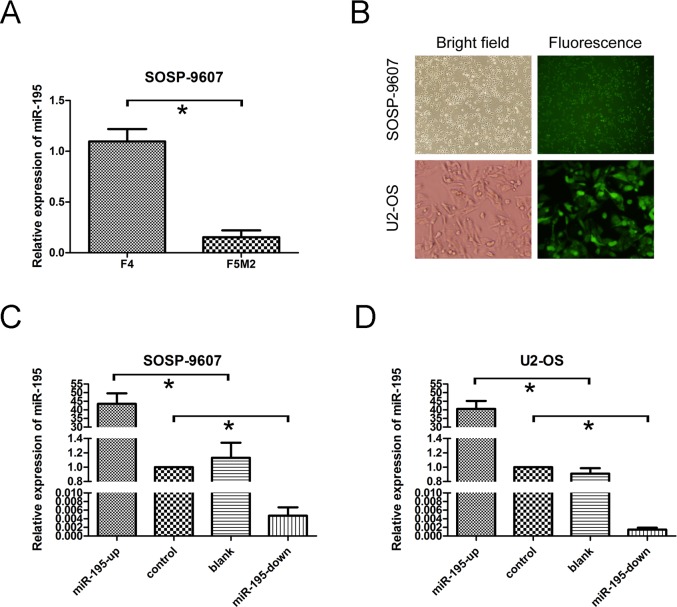
miR-195 expression and miR-195 oligonucleotides transfection in osteosarcoma cells (A) The expression of miR-195 in F4 and F5M2 cells was analyzed by real-time RT-PCR (P = 0.001).(B) SOSP-9607 cells (top) and U2-OS cells (bottom) were observed with white bright (left) and green fluorescence assay (right) in the same vision using fluorescence microscopy (100×;400×).(C) miR-195 expression levels were evaluated by real-time RT-PCR analysis in transfected SOSP-9607 cells. (D) miR-195 expression levels were assayed in transfected U2-OS cells. U6 was used as an internal loading control to normalize the results. The data were presented as the means ±SD, Columns, mean of four independent experiments; bars, SD; * P < 0.05, ** P < 0.01, *** P < 0.001.

In the present study, we established a pair of OS cell lines with different metastatic potentials named F5M2 (highly metastatic) and F4 (lowly metastatic) [19]. Gene expression and proteomic profiling were performed subsequently to identify the metastasis associated molecules [20, 21]. To further analyze the regulation mechanism regarding gene-microRNA-protein network. We applied miRNA microarray to same cell lines and generated a differential expressed miRNAs related to their extinguishable metastatic potentials. 76 differential expressing microRNAs were finally identified. (p<0.01) (Fig. 1). Among them, it is highly interested that top ranked miR-195 highly expressed in low metastatic cell lines indicating its suppressor role in metastasis (Supplementary Table 1, available at Oncotarget Online). The result of real-time RT-PCR confirmed the pattern of miR-195 expression identified by MicroRNA microarray (Fig. 2A; P<0.01).

### miR-195 inhibits migration and invasion of OS *in vitro*

**Figure 3:**
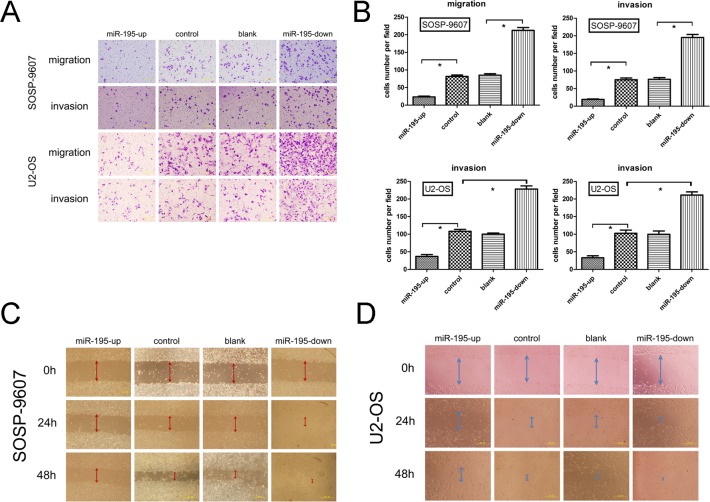
miR-195 inhibits migration and invasion of osteosarcoma *in vitro* (A) Representative photographs of migrated and invaded SOSP-9607 or U2-OS cells on the membrane at a magnification of 200×.(B) Quantitative results for the migration and invasion ability of each group of SOSP-9607 or U2-OS cells were shown as migrated and invaded cell number, 16 h after incubation. (C,D) SOSP-9607 or U2-OS cells were seeded in 6-well plates and wounds were created on the next day. Photographs were taken at hour 0, 24, and 48 h, respectively, after the wound was made. The data were presented as the means ±SD, Columns, mean of four independent experiments; bars, SD; * P < 0.05, ** P < 0.01, *** P < 0.001.

It is in controversial on the role of miR-195 in different cancer cells. The exact role of miR-195 in OS metastasis remains to be clarified. To address this question, we applied lentivirus system to make stable cell lines on the base of OS cell line SOSP-9607and U2-OS including a blank group (untransfected cells), a control group (cells transfected with the control lentivirus), an OE group (over-expression of miRNA-195) and a KD group (knocking down of miRNA-195). The miR-195 expression levels in four groups were evaluated using fluorescent microscope (Fig. 2B) and real-time RT-PCR (Fig. 2C, D). We then performed transwell migration and invasion assay to investigate the effects of miR-195 on the migratory and invasive behavior of OS cells *in vitro*. In the migration assay, the number of OE SOSP-9607 cells (23.40± 4.9295, P<0.001) were significantly lower than the KD SOSP-9607 cells (212.4 ± 18.24). No significant difference was observed between the blank and control SOSP-9607 cells (P = 0.644>0.05) (Fig. 3A, B). Invasion assay showed that the number of OE SOSP-9607 cells (19.00± 3.53, P<0.001) passing through the matrigel were significantly lower than KD SOSP-9607 cells (195.20 ± 19.54) (Fig. 3A, B). Similar results were obtained in U2-OS cell lines (Fig. 3A, B), which strongly indicated that miR-195 play a role in the reduction of migratory and invasive potential of OS *in vitro*.

Wound healing assay was used to examine the effects of miR-195 on the migration ability. Cells in OE groups exhibited an obvious decrease in migration rate as compared to the other three groups. KD SOSP-9607 (or U2-OS) cells nearly closed the wound at 48 h after incubation, whereas the other three groups were unable to close the wound at the same time point (Fig. 3C, D).

### miR-195 inhibits tumor growth and metastasis of OS *in vivo*

**Figure 4:**
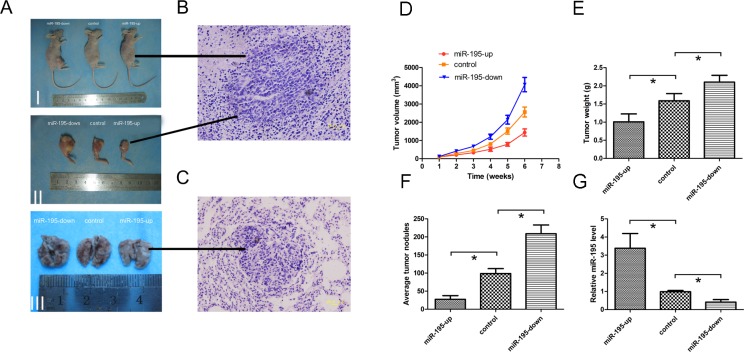
MiR-195 inhibits tumor growth and metastasis of osteosarcoma *in vivo* (A)I, Representative photographs of tumors on the right leg of mouse; II, Representative photographs of orthotopic tumors harvested 42 days after inoculation. III, Representative macroscopic pictures of mouse lungs, 42 days after inoculation. (B) Representative photographs of H&E stained spontaneous orthotopic tumors at a magnification of 400×. (C) Representative photographs of H&E stained spontaneous lung metastases at a magnification of 400×. (D) Tumor growth curves measured after the inoculation. The length (L) and width (W) of tumor measured every 7days after inoculation, and the volume of tumor was calculated according to the formula: volume = 1/2×L×W^2^. (E) Orthotopic tumor weights 42 days after inoculation. Data are presented as means±SD. (F) Graph displaying the total number of tumor nodules per lung in three groups. Data are presented as means±SD. (G) 42 days after inoculation, miR-195 expression levels in orthotopic tumors were tested and showed in relative miR-195 levels. The data were presented as the means ±SD, Columns, mean of four independent experiments; bars, SD; * P < 0.05, ** P < 0.01, *** P < 0.001.

Given these findings *in vitro*, animal studies were conducted to evaluate the effect of miR-195 on orthotopic tumor growth and metastasis in athymic nude mice. Three groups of stable cells (OE, control and KD SOSP-9607) were injected into proximal tibia of young nude mice as described in methods, respectively. The results showed that cells formed progressively growing solid tumors in all mice. By contrast, cells in OE groups produced much smaller and slowly growth tumors than the KD groups (Fig. 4A, B, p<0.05). The mean tumor weight±SD of orthotopic tumors in OE group and KD group was 1.008±0.219 g and 2.104±0.187 g, respectively (Fig. 4A, B). Lung tissue of nude mice was also removed to observe the level of metastases. The result showed that the number of metastatic node was dramatically reduced in the nude mice in OE groups when compared to the other groups (Fig. 4A, B). Then tumor and metastases were confirmed based on histopathological evaluation (Fig. 4B, C). Meanwhile, the miR-195 expression levels in the orthotopic tumors were higher in the OE groups as compared with other groups (Fig. 4G). It indicates that exogenous miR-195 can significantly inhibit the tumor growth and metastasis of OS *in vivo*.

### MiR-195 is down-regulated in human OS tissues and correlates with multiple clinical features

**Figure 5:**
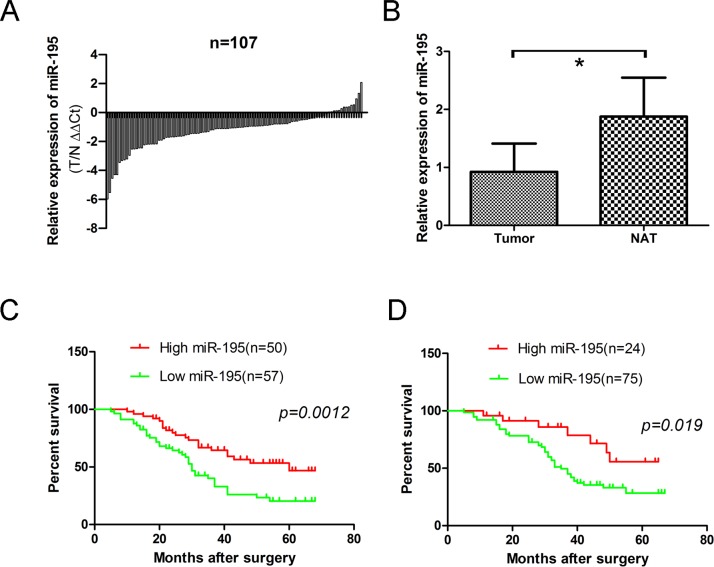
Expression level of miR-195 in 107 OS frozen samples and 99 osteosarcoma formalin- or paraformalin-fixed, paraffin-embedded (FFPE) tissues (A) Relative levels of miR-195 in 107 surgical specimens of osteosarcoma and matched adjacent noncancerous tissues(NAT) was quantified by real-time RT-PCR. Data were presented as log2 fold change (ΔΔCt values, Tumor/noncancerous tissues, T/N). (B) Means of miR-195 relative levels for 107 surgical specimens of osteosarcoma and the matched adjacent noncancerous tissues. Data were presented as 2^−ΔΔCt^ values (p=0.291>0.05). (C) Decreased expression of miR-195 was correlated with poor survival in osteosarcoma patients. Log rank tests show that patients with high miR-195 expression survived statistically significantly longer (p = 0.0012) than those with low miR-195 expression. The median miR-195 expression level (0.92479) in the tumor samples was chosen as the cut-off point. (D) Decreased expression of miR-195 was correlated with poor survival in osteosarcoma FFPE patients. Log rank tests show that patients with high miR-195 expression survived statistically significantly longer (p = 0.019) than those with low miR-195 expression. The median miR-195 expression level (5.0214) in the tumor samples was chosen as the cut-off point. The data were presented as the means ±SD, Columns, mean of four independent experiments; bars, SD; * P < 0.05, ** P < 0.01, *** P < 0.001.

To determine whether miR-195 expression correlates with clinical outcome in patients, we measured its levels in 107 pairs of OS frozen samples and corresponding noncancerous bone tissues by quantitative real time RT-PCR. Totally, miR-195 expression was decreased in 93 of 107 (86.92%) tumor samples compared with their non-malignant counterparts (Fig. 5A). The results showed that the relative level of miR-195 expression in OS tissues (mean± SD: 0.9248±0.4869) was significantly lower than that in matched nontumor adjacent tissues (NATs) (mean ± SD: 1.8762 ±0.6724; p<0.001) (Fig. 5B).

The correlations of miR-195 expression of OS tissues and clinical information were analyzed. The median (0.92479) of miR-195 expression levels in all 107 patients were used to divide the patients into lowly (n=57) and highly expression groups (n=50) [22]. We found a statistically significance between miR-195 expression and some clinical features of OS, including tumor size (p=0.020), clinical stage (p=0.0104), distant metastasis (p= 0.015) and patient mortality (p=0.006). No significant difference was observed between the expression of miR-195 and patients' age (p=0.234) and gender (p=0.576). (Table 1).

**Table 1:** Relationship between expression of miR-195, CCND1 and clinicopathologic factors in 107 OS frozen samples

		miR-195 expression			CCND1 expression		
Characteristics	NO.	Low NO.	High NO.	p	Low NO.	High NO.	p
sex				0.234			0.182
Male	62	30	32		25	37	
Female	45	27	18		24	21	
Age(years)				0.576			0.214
≥18	35	20	15		12	23	
<18	72	37	35		37	35	
Tumor size(cm2)				0.020*			0.031*
≥50	47	31	16		16	31	
<50	60	26	34		33	27	
Clinical stage				0.0104*			0.066
IIA	32	11	21		19	13	
IIB/III	75	46	29		30	45	
Distant metastasis				0.015*			0.024*
Yes	52	34	18		18	34	
No	55	23	32		31	24	
Status				0.006*			0.034*
Survival	45	17	28		26	19	
Death	62	40	22		23	39	

* Note: statistically significant P-values.

To verify what we found in frozen OS samples, we further calculated the expression levels of miR-195 in 99 OS formalin- or paraformalin-fixed, paraffin-embedded (FFPE) tissues. Similarly using its median level (5.0214) as a cutoff value, high miR-195 expression group (n=24) and low miR-195 expression group (n=75) presented a significant difference in some clinical features of OS, including tumor size (p=0.044), clinical stage (p=0.016), distant metastasis (P = 0.045) and patient mortality (p=0.038) (Table 2). No significant difference was observed between the expression of miR-195 and age (p=0.142), and patients' gender (p=0.058).

**Table 2:** Relationship between expression of miR-195 and clinicopathologic factors in 99 osteosarcoma formalin- or paraformalin-fixed, paraffin-embedded (FFPE) tissues

		miR-195 expression	
Characteristics	Number of cases	Mean±SD	p
sex			0.058
Male	55	2.1066±3.09735	
Female	44	8.9733±12.19653	
Age(years)			0.142
≥18	41	8.4747±12.80991	
<18	54	2.7768±3.71416	
Tumor size(cm2)			0.044*
≥50	36	1.8353±3.24610	
<50	63	7.6765±11.00895	
Clinical stage			0.016*
IIA	27	10.1376±13.39194	
IIB/III	72	2.4633±3.53004	
Distant metastasis			0.045*
Yes	48	1.9043±3.21113	
No	51	8.3333±11.49308	
Status			0.038*
Survival	45	8.8246±11.72106	
Death	54	1.7838±3.12864	

* Note: statistically significant P-values.

### Down-regulation of miR-195 confers poor prognosis in patients with OS

We further analyzed the correlation of miR-195 expression and overall survival of OS patients. Patients with high miR-195 expression survived statistically significantly longer than those with low miR-195 expression by using the log rank test (Fig. 5C; p=0.0012). The similar results were obtained in FFPE tissues (Fig. 5D; p=0.019).

Then we identified whether miR-195 was an independent prognostic covariate for OS, and multivariable Cox regression model showed that low levels of miR-195 expression in OS (p=0.004, relative risk =0.435) and distant metastasis (p=0.002, relative risk =1.252) were associated with a poor prognosis in terms of overall survival, independent of other clinical covariates (Table 3). The similar results were obtained in FFPE tissues (p=0.047, relative risk =0.434, Table 3)

**Table 3:** Multivariate cox regression analysis of prognostic variables in 107 OS frozen samples and OS FFPE tissues

	Variables	B	P	Wald	Relative risk	95% confidence interval
107 osteosarcoma tissues	miR-195 expression	−0.832	0.004*	8.125	0.435	0.246-0.771
	Age	0.125	0.641	0.217	1.133	0.670-1.918
	Clinical stage	0.323	0.332	0.942	1.382	0.719-2.654
	Distant metastasis	0.225	0.002*	9.368	1.252	1.084-1.446
	Tumor size(cm2)	0.227	0.394	0.727	1.255	0.745-2.114
99 FFPE tissues	miR-195 expression	−0.835	0.047*	3.933	0.434	0.19-0.99
	Age	0.26	0.337	0.920	1.297	0.762-2.209
	Clinical stage	0.256	0.428	0.627	1.292	0.685-2.438
	Distant metastasis	0.725	0.016*	5.842	2.064	1.147-3.716
	Tumor size(cm2)	0.230	0.399	0.712	1.258	0.738-2.146

### CCND1 is a potential target gene of miR-195

**Figure 6:**
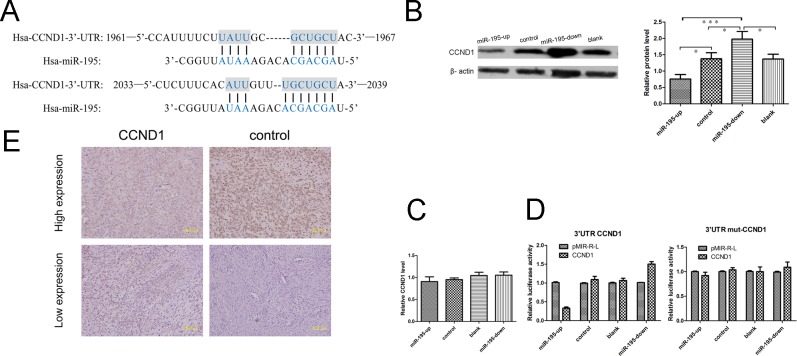
MiR-195 may target CCND1 in osteosarcoma (A) Sites of complementarity sequences between microRNAs and CCND1 mRNA. (B) The results of Western blot showed that miR-195 interacted with CCND1 and negatively regulated its expression at the translational level. (C) The results of real-time revealed that miR-195 had no effect on CCND1 in mRNA level. (D) Luciferase assays indicated that miR-195down-regulates the expression of CCND1. Relative expression of the firefly Luciferase expression was standardized for transfection control, Renilla luciferase. PMIR-REPORT™ luciferase (pMIR-R-L, Promega) was used as empty vector. All experiments were repeated three times in triplicate. Columns denotes mean of three independent experiments and bars stands for SD value. (E) Expression of Cyclin D1 was analyzed in osteosarcoma tissues with immunohistochemistry staining. The data were presented as the means ±SD, Columns, mean of four independent experiments; bars, SD; * P < 0.05, ** P < 0.01, *** P < 0.001.

To further understand the regulating mechanisms of miR-195, we examined its potential targets by searching three main-stream target prediction data bases including Pixar, miRanda and Target Scan. A conserved domain within the 3′-UTR of CCND1 with a potential miR-195 binding site was identified (Fig. 6A). To confirm this prediction, we did luciferase assay to validate CCND1 as a target of miR-195 in OS cells SOSP-9607. The miR-195-up cells significantly repressed the luciferase activity of the vector with the wild-type CCND1 3 ‘-UTR, whereas mutation of the seed sequence abolished this repression (Fig. 6D). Meanwhile, RT-PCR and Western Blot were used to detect the mRNA and protein expression level in miR-195 over-expression cell lines. The results revealed that miR-195 had no effect on CCND1 mRNA level (Fig. 6C), but we observed a clear reduction in the level of the endogenous Cyclin D1 protein in OE SOSP-9607 cells (Fig. 6B) (Supplementary Figure 1, available at Oncotarget Online). On the other hand, Cyclin D1 protein was also found highly expressed in KD SOSP-9607 cells (Fig. 6B) comparing to the control cells. These results indicate that miR-195 may target and down-regulate CCND1 expression in OS.

### Expression of the Cyclin D1 protein is reversely correlated with miR-195 expression in OS

Since Cyclin D1 has been recently found promoting metastasis of cancer cells, we wondered whether its expression mediate the anti-metastasis effect of miR-195 in OS. We examined CCND1(CyclinD1) expression in 107 OS frozen samples using real-time quantitative PCR. Of the 50 osteosarcoma cases with elevated miR-195, 30 (60.0%) showed low levels of Cyclin D1. High levels of Cyclin D1 were present in 38 of 57 (66.7%) cases with low level of miR-195 (p< 0.01) (Table 4). Then we examined the expression of Cyclin D1 in 99 paraffin specimens of OS using immunohistochemistry analysis. Representative images of Cyclin D1 were shown (Fig. 6E). Of the 24 OS cases with elevated miR-195, 17 (70.8%) had low levels of Cyclin D1. 45 of 75 (60.0%) cases with low level of miR-195 presented high level of Cyclin D1 (p<0.01) (Table 4). The expression of the Cyclin D1 protein was reversely correlated with miR-195 expression in OS.

**Table 4:** Inverse correlation of expression of miR-195 and Cyclin D1 in 107 OS frozen samples (using real-time quantitative PCR) and OS FFPE tissues(using immunohistochemistry analysis)

	Group	High miR-195		Low miR-195		In all	
107 osteosarcoma tissues	High Cyclin D1		20(40.0%)		38(66.7%)		58
	Low Cyclin D1		30(60.0%)		19(33.3%)		49
	In all		50		57		107
99 FFPE tissues	High Cyclin D1		7(29.2%)		45(60.0%)		52
	Low Cyclin D1		17(70.8%)		30(40.0%)		47
	In all		24		75		99

To gain an insight into the role of Cyclin D1 in human OS development, we analyzed the association of the expression of Cyclin D1 with clinicopathological parameters in 107 OS patients. As shown in Table 1, the expression of Cyclin D1 was positively associated with tumor size (p = 0.031), distant metastasis (p = 0.024) and poor survival (p = 0.034). These highly correlated expression patterns substantially suggested the potential role of CCND1being the target of miR-195 in OS.

## DISCUSSION

OS is the most common bone primary malignancy in adolescents [23]. With the advancement of neoadjuvent chemotherapy in 1970s', the global survival rate of newly diagnosis cases was improved to about 70% and remains for three decades [24]. However, like most malignant cancers, the occurrence of metastasis still result in highly mortality rate. It is reported the patients with initial metastasis at diagnosis only had 30% survival chances which pose a huge threat to the global survival of OS patients [2]. One of the major reasons hampering the current regiments lies in the unknown mechanisms of metastasis and rare target molecules available. Our Lab has been contributing to identify the metastasis molecules of OS using high throughout genomics and proteomics methods. In the previous studies, based on a unique pair of OS cell lines with differential potentials, we succeed in identifying metastasis related genes and proteins. During the data analysis, we found a lot of discrepancy phenomenon between two data sets which intrigued a hypothesis there were other regulating mechanisms than translation involving this course. MicroRNAs has been widely explored for their regulating function in different expression levels, such as transcription, translation and even degradation [25]. In present study, we generated a OS metastasis related miRNA profiling and found one of the top ranked miRNAs miR-195 inhibit the cancer metastasis *in vivo and in vitro* partly through targeting the cell cycle gene-CCND1(CyclinD1). It is clear that CCND1 encodes CyclinD1 protein [26] and the new discoveries of CCND1 on tumor metastasis are novel and attractive [15]. It is especially interested that in previous study cyclinD1 is up-regulated in highly metastatic F5M2 proteomics profiling data while the gene expression level was found down-expressed [20, 21]. The lowly expressed regulating microRNAs such as miR-195 might mediate the up-regulation of cyclinD1 in this context. More integrating analysis on these omics data generating from the different platforms need to be further performed.

MiR-195, one of the miR-16/15/195/424/497 family members, has been found aberrantly deregulated in tumorigenesis [12]. For example, miR-195 is up-regulated in chronic and acutely mphocytic leukemia [10] and melanoma [11] but down-regulated in adrenocortical, hepatocellular carcinoma, and squamous cell carcinoma [12-14]. These controversial observations indicate the complexity of miR-195 during tumorgenesis and development and also call for the further explore of the role in the metastasis of OS. In our study, we showed here that over-expression of miR-195 significantly suppressed the migration and invasive nature of SOSP-9607 cells and U2-OS cells *in vitro*. Also, a mouse study further proved the inhibitory effects of miR-195 on tumor pulmonary metastasis. More importantly, using 107 pairs of in-house samples, we confirmed the miR-195 frequently down-regulated in OS tissues comparing to their adjacent normal tissue. In addition, using median expression level as a cut-off value, miR-195 was shown to stratify patient survival indicating its potential as a biomarker for metastasis. However, additional study to establish a standard procedure needs to be achieved.

Over the last decade it has become increasingly clear that Cyclins and Cyclin-binding proteins have cell cycle-independent roles. Recent studies suggest Cyclin D1 also plays a key role in promoting cellular migra­tion of epithelial cells, which shows the new roles of Cyclin D1 in tumor progression and metastasis [15]. Cyclin D1 has a function in transcriptional control that does not involve CDKs which involves promoter recruitment of histone deacetylases (HDACs) and histone methyltransferases including SUV39 and HP1 [27]. Our results in OS cell lines indicate that miR-195 targets Cyclin D1 and negatively regulates its expression at the translational level but not in mRNA level which indicates that miR-195 may suppress metastasis in OS cells by down-regulating CCND1 oncogene.

Unlike siRNAs which silence the expression of a single gene, miRNAs mainly silence the expression of multiple genes simultaneously. It is likely that miR-195 may also regulate other genes beside CCND1. Therefore, it is critical to identify more target genes that mediate the miR-195 induced regulation of metastasis. By using TargetScan, PicTar and miRanda, we predicted putative genes of miR-195 which had not been experimentally identified yet, and finally obtained several putative targets which were reported correlating with tumor growth or metastasis, such as FBXL20, POU2F1, PPPDE2, SCAI, which need further analysis. Further investigations are also required for characterization of the interaction of miR-195 and these target genes separately and synergistically in OS.

In conclusion, the results presented here demonstrate that miR-195 has great biological effects on the metastasis of OS cells both *in vitro* and *in vivo*. Over-expression of miR-195 down-regulate the expression of Cyclin D1 protein, suggesting that miR-195 functions as tumor suppressors probably through down-regulating CCND1 in OS. Down-regulation of miR-195 may be associated with tumor aggressiveness and tumor metastasis of OS, which means miR-195 may be an independent prognostic marker for OS patients. Furthermore, there are other putative miR-195 target genes which could potentially be key players in the metastasis of OS cells. The miR-195 may prove to be a promising gene therapeutic agent.

## MATERIALS AND METHODS

### Ethics statement

All research involving human tissue samples and animals was approved by the Ethics Review Committee of Fourth Military Medical University, Xi'an, Shaanxi, China (approval ID:2013107) and written informed consent was obtained from all participating patients.

### Human tissue samples

A total of 107 pairs of human OS tissue samples were obtained from patients who underwent surgical resection at the Tang Du Hospital of Fourth Military Medical University between 2007 and 2010 and were diagnosed with OS based on histopathological evaluation. The biopsies were immediately snap-frozen in liquid nitrogen after resection and stored at −80°C. One section of each sample was stained with hematoxylin-eosin (H&E) for histopathological evaluation. The clinical stage of these OS patients was classified according to the sixth edition of the tumor–node–metastases (TNM) classification of the International Union Against Cancer (UICC).

All 107 OS patients received follow-up. The median follow-up was 42 months (range 5–68 months). During the follow-up period, 62 patients (57.9%) died of disease. Distant metastases developed in 52 patients at a mean of 12.7 months (range 3–41 months) after the original diagnosis and among them 13 had bone metastases and 43 had lung metastases (4 patients had both bone and lung metastases).

### Cell culture

Human OS cell lines SOSP-9607 ware established and reserved in our laboratory as described previously [19]. Human OS cell lines U2-OS was purchased from ATCC.SOSP-9607 cells were maintained in RPMI 1640 medium (HyClone, USA) supplemented with 10% fetal bovine serum (FBS; HyClone), 2.0mM l-glutamine, 100U/ml penicillin, and 100ug/ml streptomycin, and incubated at 37°C in a humidified incubator supplemented with 5% CO_2_ and 95% air. U2-OS cells were maintained in the same conditions, except DMEM medium was used.

### F5M2 and F4

F4 and F5 were the sublines originated from SOSP-9607 using limited dilution method [28]. Briefly, SOSP-9607 was harvested with 2.5g/L trypsin and diluted gradient to the final density 230cells/4.6ml and seeded at 0.1ml/well in 36 wells of 96-well plates. The remain volume was added to 5ml and seeded again in the other 36 wells. The last 1.4ml medium was diluted to 2.8ml and then was transported to the last 24 wells at 0.1ml/well. The single cell well was identified under the microscope by three individuals and amplified cultured. F5M2 was derived by intra-osseous injection of the F5 cells which showed the higher pulmonary metastasis potential to the proximal tibia of a nude mice using method previously reported [19]. The resultant tumor in local site grew quickly and developed spontaneous metastasis to lung after transplantation in less than 5 weeks. The metastasis nodules were removed from the lung and enzymaticly digested to a single cell suspension, propagated and maintained *in vitro* which was named F5M1. Then F5M1 was implanted to the tibia of mice again 6 weeks later. The metastasis nodules were mechanically and enzymatically digested to a single cell suspension, propagated and maintained *in vitro* which was named F5M2. F4 was the clone that showed the lower pulmonary metastasis ability confirmed by tumor cell orthotopic transplantation assay scribed above.

### MicroRNA microarray assay

Total RNA was extracted by using a FastTrack 2.0 mRNA isolation kit (Invitrogen, California) according to the supplier's protocol. Microarray analysis was performed using LC MicroRNA microarray Chip_H11.0_081344 which detects miRNA transcripts listed in Sanger miRBase Release 11.0. Imaging and quantifying analysis were performed using Affymetrix scanner and a fag software.

### Generation of stable cell lines

Recombinant lentiviruses containing over-expression of miRNA-195, knocking down of miRNA-195 and miRNA control were purchased from GeneChem (Shanghai, China). Besides the multiple clone sites of lenti-virus expression vectors, there also was a GFP reporter driven by an independent promoter (SV40 promoter) to indicate the infection rate of virus infection timely.

To generate the stable cell line, 1×10^4^ cells were transfected with 5×10^5^ transducing units of lentiviruses. The supernatant was removed after 24h and replaced with complete culture medium. Infection efficiency was confirmed by RT-PCR 96h after infection and the cells were selected with 1μg/ml puromycin for 2 weeks.

### Migration and invasion assays

The invasive potential of cells was measured in 6.5 mm Transwell with 8.0 mm Pore Polycarbonate Membrane Insert (Corning, NY) according to the manufacturer's instructions. The filter of top chamber was matrigel-coated with 50 μl of diluted matrigel and incubated at 37°C for 2 h. The lower chambers were filled with 600 μl of RPMI-1640 medium containing 5% fetal bovine serum(FBS) as chemoattractant. Cells were serum-free-starved overnight, and harvested and resuspended in migration medium (RPMI-1640 with 0.5% BSA). The suspension of 5,000 cells in 100 μl of migration medium was added into each top chamber. After the cells were incubated for 16 h, the non-invading cells that remained on the upper surface were removed by using a cotton swab. The invasive cells on the lower surface of the membrane insert were fixed with 4% paraformaldehyde for 30 min, permeabilized with 0.2% Triton X-100 at room temperature for 15 min, and then stained with 0.1% crystal violet for 5 min. The number of cells on the lower surface, which had invaded through the membrane, was counted under a light microscope in five random fields at a magnification of 100×. The experiments were repeated thrice independently and results were given as means±SD.

The procedure for transwell migration assays were the same as the transwell invasion assay except that the filter of top chamber was not coated with matrigel.

### Wound healing migration assay

When the transfected and untransfected SOSP-9607 and U2-OS cells were grown to confluence, a scratch in the cell monolayer was made with a micropipette tip. Following incubation of the cells under standard conditions for 24 h, the plates were washed twice with fresh medium and images were captured at different times. The migration potential was estimated by counting the cells that migrated from the wound edge. The cell migration rate was obtained by counting three fields per area and represented as the average of six independent experiments over multiple days.

### Animal studies

Four-week-old female nude mice (BALB/c, nu/nu; animal center of the Fourth Military Medical University in China(FMMU)), 17.0g–22.0g in weight, were maintained under specific pathogen-free conditions with 12-h light/12-h dark cycle at 26–28°C and 50–65% humidity. Animal feed and underpad, which were purchased from Experimental Animal Center of Fourth Military Medical University, were autoclaved and vacuum packed. The water was adjusted to the pH-value of 2.8 and autoclaved before use.

Animal experiment was performed to evaluate orthotopic tumor growth and spontaneous pulmonary metastasis properties of OS cells *in vivo*. In brief, 3 groups SOSP-9607cells (over-expression of miRNA-195, knocking down of miRNA-195 and miRNA control cells) were harvested by treatment with trypsin-EDTA (Invitrogen), washed twice with PBS, and resuspended in PBS. Then OS cells suspension of 100,000 cells in 100 μl were injected into the proximal tibia of each anesthetized nude mice (n = 10 animals/group). Every 7days post inoculation, the length and width of individual orthotopic tumor from each mouse were measured with calipers, and the volume (mm^3^) of orthotopic tumor was calculated according to the formula: ½ × length × width^2^ [29]. The curve of orthotopic tumor growth was depicted 42 days after inoculation, and mouse lungs and orthotopic tumors were harvested. The orthotopic tumors were weighed, the miR-195 expression levels in the orthotopic tumors were tested by real time RT-PCR, and the number of pulmonary metastatic tumor nodules was counted under a low-powered dissecting stereomicroscope. Finally, mouse lungs were fixed with 10% neutral-buffered formalin, embedded in paraffin, sectioned at 6 μm and stained with H&E (hematoxylin and eosin). The pulmonary metastases were imaged under a light microscope at a magnification of 40×, 100×, 200× and 400×.

### Quantitative real-time RT-PCR

Total RNA containing miRNA and mRNA was extracted from cells with Trizol Reagent (Invitrogen), or from formalin or paraformalin-fixed,paraffin-embedded (FFPE) tissues with Recover All TM Total Nucleic Acid Isolation Kit (Ambion, USA), according to the manufacturer's instructions. The RNA was transcribed into cDNA using BioRT Two Step RT-PCR kit (Bioer). For evaluating the Cyclin D1 expression levels, 1 μg of total RNA was used for reverse transcription with iScript cDNA Synthesis Kit (Bio-Rad, USA) according to the manufacturer's instructions. Quantitative real-time RT-PCR was performed with iQ SYBR Green Supermix (Bio-Rad, USA) according to the manufacturer's instructions on real-time Instrument ABI-PRISM 7000 (Applied Biosystems, USA). The sequences of the forward and reverse primers for CCND1 were 5′-CTGTCCCACTCCTACGATACG-3′ and 5′-CAGCATCTCATAAACAGGTCACTAC-3′, respectively. The sequences of the forward and reverse primers for glyceraldehyde-3-phosphate dehydrogenase (GAPDH) were 5′-GGGTGTGAACCATGAGAAGT-3′ and 5′-TGAGTCCTT CCACGATACCAA-3′, respectively. GAPDH was used as a control. To evaluate has-mir-195 levels, the sequences of primers for miR-195: 5′-ACACTCCAGCTGGGTAGCAGCACAGAAAT-3′ were used.U6 was used as a control.

### Protein extraction and western blotting analysis

Protein extracts from cells were prepared through a modified RIPA buffer with 0.5% sodium dodecyl sulfate (SDS) in the presence of a proteinase inhibitor cocktail (Complete Mini; Roche Diagnostics, Switzerland) and then were performed as described previously [30]. The samples derived from the same experiment and that blots were processed in parallel.

### Luciferase reporter assay

For validation of CCND1 as a target gene of miR-195 in OS cells, luciferase assay was performed as described previously [31].

### Target prediction

Bioinformatics analysis was performed by using specific programs: Pictar (http://pictar.mdc-berlin.de/), miRanda (http://www.microrna.org) and TargetScan (http://www.targetscan.org/).

### Immunohistochemistry

The dilution of Cyclin D1 antibody used for immunohistochemistry were 1:100. Immunohistochemistry were performed as described previously [32]. The final scores of Cyclin D1 expression were calculated as described previously [33] and classified as follows [33]: 0–4 as low; 5–9 as high.

### Statistical analysis

All values in the paper were expressed as the means ± SD, and all error bars represent the standard deviation of the mean. Student's *t* test, one-way analysis of variance and repeated measures data of ANOVA were used to determine significance. Patient survival and their differences were determined using the log rank test. Cox regression (proportional hazards model) was used for multivariate analysis of prognostic factors. All statistical tests were two-sided. p<0.05 was considered statistically significant. Statistical analyses were performed using SPSS 17.0 software (SPSS Inc, USA).

## SUPPLEMENTARY MATERIALS, FIGURES AND TABLES



## References

[1] Mirabello L, Troisi RJ, Savage SA (2009). Osteosarcoma incidence and survival rates from 1973 to 2004: data from the Surveillance, Epidemiology, and End Results Program. Cancer.

[2] Chou AJ, Geller DS, Gorlick R (2008). Therapy for osteosarcoma: where do we go from here?. Paediatric drugs.

[3] Bartel DP (2004). MicroRNAs: genomics, biogenesis, mechanism, and function. Cell.

[4] Kim VN (2005). MicroRNA biogenesis: coordinated cropping and dicing. Nature reviews Molecular cell biology.

[5] Filipowicz W, Jaskiewicz L, Kolb FA, Pillai RS (2005). Post-transcriptional gene silencing by siRNAs and miRNAs. Current opinion in structural biology.

[6] Ambros V (2001). microRNAs: tiny regulators with great potential. Cell.

[7] He L, Hannon GJ (2004). MicroRNAs: small RNAs with a big role in gene regulation. Nature reviews Genetics.

[8] Lagos-Quintana M, Rauhut R, Meyer J, Borkhardt A, Tuschl T (2003). New microRNAs from mouse and human. RNA (New York, NY).

[9] Landgraf P, Rusu M, Sheridan R, Sewer A, Iovino N, Aravin A, Pfeffer S, Rice A, Kamphorst AO, Landthaler M, Lin C, Socci ND, Hermida L, Fulci V, Chiaretti S, Foa R (2007). A mammalian microRNA expression atlas based on small RNA library sequencing. Cell.

[10] Zanette DL, Rivadavia F, Molfetta GA, Barbuzano FG, Proto-Siqueira R, Silva-Jr WA, Falcao RP, Zago MA (2007). miRNA expression profiles in chronic lymphocytic and acute lymphocytic leukemia. Brazilian journal of medical and biological research = Revista brasileira de pesquisas medicas e biologicas / Sociedade Brasileira de Biofisica [et al].

[11] Bhattacharya A, Schmitz U, Wolkenhauer O, Schonherr M, Raatz Y, Kunz M (2013). Regulation of cell cycle checkpoint kinase WEE1 by miR-195 in malignant melanoma. Oncogene.

[12] Wang X, Wang J, Ma H, Zhang J, Zhou X (2012). Downregulation of miR-195 correlates with lymph node metastasis and poor prognosis in colorectal cancer. Medical oncology (Northwood, London, England).

[13] Xu T, Zhu Y, Xiong Y, Ge YY, Yun JP, Zhuang SM (2009). MicroRNA-195 suppresses tumorigenicity and regulates G1/S transition of human hepatocellular carcinoma cells. Hepatology (Baltimore, Md).

[14] Soon PS, Tacon LJ, Gill AJ, Bambach CP, Sywak MS, Campbell PR, Yeh MW, Wong SG, Clifton-Bligh RJ, Robinson BG, Sidhu SB (2009). miR-195 and miR-483-5p Identified as Predictors of Poor Prognosis in Adrenocortical Cancer. Clinical cancer research: an official journal of the American Association for Cancer Research.

[15] Li Z, Wang C, Prendergast GC, Pestell RG (2006). Cyclin D1 functions in cell migration. Cell cycle (Georgetown, Tex).

[16] Arnold A, Papanikolaou A (2005). Cyclin D1 in breast cancer pathogenesis. Journal of clinical oncology: official journal of the American Society of Clinical Oncology.

[17] Fu M, Wang C, Li Z, Sakamaki T, Pestell RG (2004). Minireview: Cyclin D1: normal and abnormal functions. Endocrinology.

[18] van Diest PJ, Michalides RJ, Jannink L, van der Valk P, Peterse HL, de Jong JS, Meijer CJ, Baak JP (1997). Cyclin D1 expression in invasive breast cancer. Correlations and prognostic value. The American journal of pathology.

[19] Chen X, Yang TT, Wang W, Sun HH, Ma BA, Li CX, Ma Q, Yu Z, Fan QY (2009). Establishment and characterization of human osteosarcoma cell lines with different pulmonary metastatic potentials. Cytotechnology.

[20] Chen X, Yang TT, Zhou Y, Wang W, Qiu XC, Gao J, Li CX, Long H, Ma BA, Ma Q, Zhang XZ, Yang LJ, Fan QY (2014). Proteomic profiling of osteosarcoma cells identifies ALDOA and SULT1A3 as negative survival markers of human osteosarcoma. Molecular carcinogenesis.

[21] Chen X, Yang TT, Qiu XC, Ji ZG, Li CX, Long H, Zhou Y, Ma BA, Ma Q, Zhang X, Fan QY (2011). Gene expression profiles of human osteosarcoma cell sublines with different pulmonary metastatic potentials. Cancer biology & therapy.

[22] Wang R, Zhao N, Li S, Fang JH, Chen MX, Yang J, Jia WH, Yuan Y, Zhuang SM (2013). MicroRNA-195 suppresses angiogenesis and metastasis of hepatocellular carcinoma by inhibiting the expression of VEGF, VAV2, and CDC42. Hepatology (Baltimore, Md).

[23] Arndt CA, Rose PS, Folpe AL, Laack NN (2012). Common musculoskeletal tumors of childhood and adolescence. Mayo Clinic proceedings Mayo Clinic.

[24] Luetke A, Meyers PA, Lewis I, Juergens H (2014). Osteosarcoma treatment - where do we stand? A state of the art review. Cancer treatment reviews.

[25] Morozova N, Zinovyev A, Nonne N, Pritchard LL, Gorban AN, Harel-Bellan A (2012). Kinetic signatures of microRNA modes of action. RNA (New York, NY).

[26] Simpson JF, Quan DE, O'Malley F, Odom-Maryon T, Clarke PE (1997). Amplification of CCND1 and expression of its protein product, cyclin D1, in ductal carcinoma in situ of the breast. The American journal of pathology.

[27] Fu M, Rao M, Bouras T, Wang C, Wu K, Zhang X, Li Z, Yao TP, Pestell RG (2005). Cyclin D1 inhibits peroxisome proliferator-activated receptor gamma-mediated adipogenesis through histone deacetylase recruitment. The Journal of biological chemistry.

[28] Grenman R, Burk D, Virolainen E, Buick RN, Church J, Schwartz DR, Carey TE (1989). Clonogenic cell assay for anchorage-dependent squamous carcinoma cell lines using limiting dilution. International journal of cancer Journal international du cancer.

[29] Naito S, von Eschenbach AC, Giavazzi R, Fidler IJ (1986). Growth and metastasis of tumor cells isolated from a human renal cell carcinoma implanted into different organs of nude mice. Cancer research.

[30] Pan Z, Zhao W, Zhang X, Wang B, Wang J, Sun X, Liu X, Feng S, Yang B, Lu Y (2011). Scutellarin alleviates interstitial fibrosis and cardiac dysfunction of infarct rats by inhibiting TGFbeta1 expression and activation of p38-MAPK and ERK1/2. British journal of pharmacology.

[31] Zhao G, Cai C, Yang T, Qiu X, Liao B, Li W, Ji Z, Zhao J, Zhao H, Guo M, Ma Q, Xiao C, Fan Q, Ma B (2013). MicroRNA-221 induces cell survival and cisplatin resistance through PI3K/Akt pathway in human osteosarcoma. PloS one.

[32] Osaki M, Takeshita F, Sugimoto Y, Kosaka N, Yamamoto Y, Yoshioka Y, Kobayashi E, Yamada T, Kawai A, Inoue T, Ito H, Oshimura M, Ochiya T (2011). MicroRNA-143 regulates human osteosarcoma metastasis by regulating matrix metalloprotease-13 expression. Molecular therapy: the journal of the American Society of Gene Therapy.

[33] Kong W, He L, Coppola M, Guo J, Esposito NN, Coppola D, Cheng JQ (2010). MicroRNA-155 regulates cell survival, growth, and chemosensitivity by targeting FOXO3a in breast cancer. The Journal of biological chemistry.

