# Supplementary material for: MicroRNA profiling identifies MiR-195 suppresses osteosarcoma cell metastasis by targeting CCND1
Source: Oncotarget. 2015 Mar 12;6(11):8875–89. doi: 10.18632/oncotarget.3560 (PMC4496189; doi:10.18632/oncotarget.3560)
Supplement: Supplementary file 1 [file oncotarget-06-8875-s001.pdf]

MicroRNA profiling identifies MiR-195 suppresses osteosarcoma cell metastasis by targeting CCND1

Supplementary Material

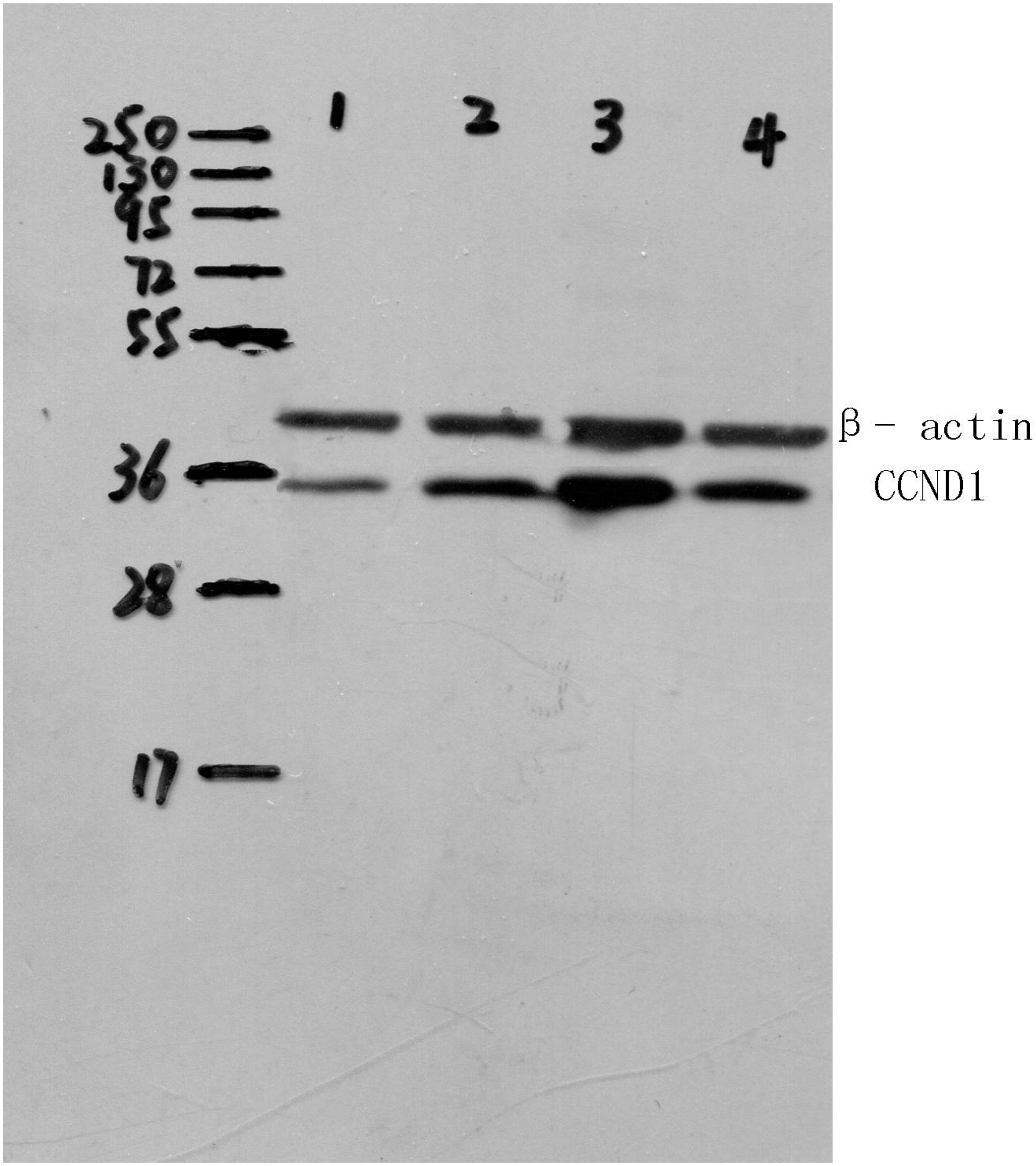

Supplementary Figure 1: Western blot showed that miR-195 interacted with CCND1 and

negatively regulated its expression (CyclinD1)at the translational level. (1) OE groups.(2) control groups. (3) KD groups. (4) blank groups. The samples derived from the same experiment and that blots were processed in parallel. The data were presented as the means ±SD, Columns, mean of four independent experiments; bars, SD; * P < 0.05, ** P < 0.01, *** P < 0.001.

Supplementary Table 1: Differently expressed transcripts with p-value<0.01.

**NO.**	**Probe_ID**	**F4 group singal**	**F5 group singal**	**Log2(F4/F5)**
1	Has-miR-192	50.78	608.14	3.66
2	Has-miR-936	3,331.72	533.13	-2.63
3	Has-miR-195	3,961.45	772.93	-2.37
4	Has-miR-886-3p	30.04	135.54	2.18
5	Has-miR-145	286.34	79.91	-1.84
6	Has-miR-1308	7,787.88	2,515.09	-1.63
7	Has-miR-196b	4,627.91	1,498.74	-1.62
8	Has-miR-194	71.54	219.06	1.56
9	Has-let-7d	158.02	456.54	1.48
10	Has-miR-483-5p	650.35	244.00	-1.42
11	Has-miR-615-3p	72.16	176.12	1.26
12	Has-miR-335	158.24	330.17	1.06
13	Has-miR-1260	290.55	147.82	-0.97
14	Has-miR-148a	322.69	615.13	0.91
15	Has-miR-30b	6,795.27	3,603.37	-0.89
16	Has-miR-148b	1,126.72	2,239.75	0.88
17	Has-miR-193b	916.43	1,567.96	0.86
18	Has-miR-15a	332.10	600.81	0.85
19	Has-miR-1275	666.18	367.17	-0.84
20	Has-miR-30e	868.03	483.79	-0.82
21	Has-miR-1275	2,97.79	1,430.87	-0.80
22	Has-miR-30e	264.52	451.63	0.79
23	Has-miR-152	699.62	1,111.78	0.75
24	Has-miR-197	837.41	514.80	-0.70
25	Has-miR-16	12,579.37	7,750.79	-0.70
26	Has-miR-181d	614.68	980.23	0.69
27	Has-miR-320d	4,791.77	7,567.90	0.68
28	Has-miR-374b	3,700.43	2,346.49	-0.67
29	Has-let-10b	1,749.30	2,765.26	0.66
30	Has-miR-224	2,380.03	3,635.34	0.64
31	Has-miR-194	3,334.86	5,234.35	0.63
32	Has-miR-320e	5,827.66	8,734.71	0.62
33	Has-miR-30e	622.95	414.21	-0.59
34	Has-miR-130a	273.89	416.96	0.59
35	Has-miR-10a	2,997.47	4,573.96	0.59

36	Has-miR-19b	1,962.20	1,306.87	-0.59
37	Has-miR-98	11,996.48	8,018.20	-0.58
38	Has-miR-181b	792.81	1,181.14	0.57
39	Has-miR-1290	970.71	1,433.34	0.57
40	Has-miR-17	4,430.48	6,526.95	0.55
41	Has-miR-30c	7,063.90	4,779.28	-0.54
42	Has-miR-1268	788.63	1,137.77	0.52
43	Has-miR-320b	6,328.87	9,007.04	0.52
44	Has-miR-320c	6,456.88	9,219.90	0.51
45	Has-miR-106a	4,379.65	6,420.84	0.51
46	Has-miR-185	508.14	710.14	0.48
47	Has-miR-25	14,614.14	10,221.98	-0.47
48	Has-let-30d	1,138.87	1,454.60	0.45
49	Has-miR-768-5p	915.68	673.60	-0.44
50	Has-miR-27b	4,373.43	5,900.75	0.43
51	Has-miR-183	911.70	1,231.75	0.43
52	Has-miR-23b	16,751.37	21,800.87	0.43
53	Has-miR-20a	5,679.21	7,481.09	0.42
54	Has-let-7i	8,904.44	11,954.81	0.42
55	Has-miR-23a	18,039.08	22,961.76	0.41
56	Has-miR-27a	5,715.73	7,623.16	0.41
57	Has-miR-30a	1,009.51	768.14	-0.40
58	Has-miR-423-5p	2,427.59	1,922.14	-0.34
59	Has-miR-29a	3,324.53	4,174.07	0.33
60	Has-miR-20b	3,298.54	4,327.94	0.32
61	Has-miR-100	3,703.15	4,697.82	0.32
62	Has-miR-151-5p	4,159.76	3,463.78	-0.30
63	Has-miR-365	3,792.26	4,609.45	0.28
64	Has-miR-21	29,393.31	36,137.54	0.28
65	Has-miR-182	4,943.58	4,171.80	-0.24
66	Has-miR-25b	8,178.23	6,974.90	-0.24
67	Has-miR-26b	7,235.18	6,221.37	-0.22
68	Has-miR-923	4,543.07	3,822.11	-0.20
69	Has-let-7e	23,373.80	20,447.28	-0.19
70	Has-miR-196a	13,967.28	12,417.03	-0.18
71	Has-miR-24	4,803.44	5,470.98	0.18
72	Has-miR-1246	13,368.56	14,909.48	0.16
73	Has-let-7b	22,970.47	20,879.19	-0.15
74	Has-miR-26a	13,107.75	12,172.31	-0.11
75	Has-miR-92b	12,673.54	11,745.59	-0.10
76	Has-let-7d	26,334.70	24,651.30	-0.08
